# From IMGT-ONTOLOGY to IMGT/LIGMotif: the IMGT^® ^standardized approach for immunoglobulin and T cell receptor gene identification and description in large genomic sequences

**DOI:** 10.1186/1471-2105-11-223

**Published:** 2010-04-30

**Authors:** Jérôme Lane, Patrice Duroux, Marie-Paule Lefranc

**Affiliations:** 1 IMGT®, the international ImMunoGeneTics information system®, Université Montpellier 2, Laboratoire d'ImmunoGénétique Moléculaire LIGM, UPR CNRS 1142, Institut de Génétique Humaine IGH, 141 rue de la Cardonille, 34396 Montpellier cedex 5, France; 2 Institut Universitaire de France, 103 Bd St Michel, 75005 Paris, France

## Abstract

**Background:**

The antigen receptors, immunoglobulins (IG) and T cell receptors (TR), are specific molecular components of the adaptive immune response of vertebrates. Their genes are organized in the genome in several loci (7 in humans) that comprise different gene types: variable (V), diversity (D), joining (J) and constant (C) genes. Synthesis of the IG and TR proteins requires rearrangements of V and J, or V, D and J genes at the DNA level, followed by the splicing at the RNA level of the rearranged V-J and V-D-J genes to C genes. Owing to the particularities of IG and TR gene structures related to these molecular mechanisms, conventional bioinformatic software and tools are not adapted to the identification and description of IG and TR genes in large genomic sequences. In order to answer that need, IMGT^®^, the international ImMunoGeneTics information system^®^, has developed IMGT/LIGMotif, a tool for IG and TR gene annotation. This tool is based on standardized rules defined in IMGT-ONTOLOGY, the first ontology in immunogenetics and immunoinformatics.

**Results:**

IMGT/LIGMotif currently annotates human and mouse IG and TR loci in large genomic sequences. The annotation includes gene identification and orientation on DNA strand, description of the V, D and J genes by assigning IMGT^® ^labels, gene functionality, and finally, gene delimitation and cluster assembly. IMGT/LIGMotif analyses sequences up to 2.5 megabase pairs and can analyse them in batch files.

**Conclusions:**

IMGT/LIGMotif is currently used by the IMGT^® ^biocurators to annotate, in a first step, IG and TR genomic sequences of human and mouse in new haplotypes and those of closely related species, nonhuman primates and rat, respectively. In a next step, and following enrichment of its reference databases, IMGT/LIGMotif will be used to annotate IG and TR of more distantly related vertebrate species. IMGT/LIGMotif is available at http://www.imgt.org/ligmotif/.

## Background

The immune adaptive system defends multicellular organisms from pathogens (i.e. bacteria, parasites, viruses) and tumor cells which are specifically recognized by antigen receptors. These antigen receptors, immunoglobulins (IG) or antibodies [1] and T cell receptors (TR) [2], present a huge diversity (2.10^12 ^IG and 2.10^12 ^TR per individual) that is crucial for specific antigen recognition. These huge numbers of different proteins are encoded by a relatively limited number of genes organized in the genome in different loci (7 in humans) that comprise different types of gene: variable (V), diversity (D), joining (J) and constant (C) genes. Synthesis of the IG and TR proteins requires complex mechanisms that include, at the DNA level, rearrangements of V and J, or of V, D and J genes [3], N-Diversity at the resulting V-J and V-D-J junctions [4,5] and, for the IG, somatic hypermutations [6,7]. These rearrangements are followed, at the RNA level, by the splicing of rearranged V-J and V-D-J genes to a C gene. In order to manage IG and TR data, IMGT^®^, the international ImMunoGeneTics information system^®^, http://www.imgt.org/[8] was created in 1989, by the Laboratoire d'ImmunoGénétique Moléculaire LIGM (Université Montpellier 2 and CNRS). One of the first goals of IMGT^® ^was to identify and to describe all the human IG and TR genes present in the human genome, an indispensable requisite before analysing the immune repertoire. Owing to the particularities of the IG and TR gene structures, IMGT-ONTOLOGY [9-11], the first ontology for immunogenetics and immunoinformatics, has been built to ensure the accuracy and the consistency of the IMGT^® ^data, as well as the coherence between the IMGT^® ^databases, tools and Web resources [12]. Several years of expert and time consuming manual curation led to the IMGT^® ^gene nomenclature for IG and TR genes [1,2] which was approved by the Human Genome Organisation (HUGO) Nomenclature Committee (HGNC) in 1999 [13] and by the World Health Organization-International Union of Immunological Societies (WHO-IUIS) [14,15]. IMGT^® ^IG and TR genes have been entered in IMGT/GENE-DB [16], the IMGT^® ^gene database, in the Human Genome Database (GDB) [17], in LocusLink [18] at the National Center for Biotechnology Information (NCBI), in Entrez Gene [19] when this database superseded LocusLink, in Ensembl [20] at the European Bioinformatics Institute (EBI), and in the Vega Genome Browser [21] at the Wellcome Trust Sanger Institute.

Interestingly, the human IG and TR genes data were annotated in IMGT^® ^[1,2] before the release of the human genome sequence [22,23], however most of the corresponding genomic sequences were short (1-2 kb) and large contigs still remain to be precisely annotated. Conventional software and tools such as GeneMark [24], Genescan [25] and N-SCAN [26], are not adapted to the annotation of IG and TR genes owing to the particularities of their structure. Prediction of immunoglobulin superfamily protein genes with Exegesis [27], a procedure which uses GeneWise [28] and experimental maps, showed improvement by comparison with the Ensembl method. However, this procedure has not been developed for detailed and standardized annotation. To answer the need of a tool for an automated annotation of antigen receptors in genomic DNA and, thus, to avoid several manual and time consuming steps, IMGT/LIGMotif, a Java on-line software has been developed, that allows the identification, standardized description and functionality assignment of IG and TR V, D and J genes in large genomic sequences.

## Methods

### IG and TR gene characteristics to consider for gene identification

IG and TR V, D and J genes belong to multigene subgroups and, therefore, share a high percentage of sequence identity (>75%) as a result of gene duplications inside a given subgroup [1,2]. As a consequence it is often difficult to assign nearly identical sequences either to different genes or to different alleles of a same gene. This can lead to possible errors in the genome assembly that need to be detected.

IG and TR loci contain several hundreds of genes, 608-665 IG and TR genes per haploid genome in human, depending on the haplotypes, and more than 800 IG and TR genes in mouse [16], these numbers including many pseudogenes (227-253 in human and 212-240 in mouse). Many of these pseudogenes are degenerated and/or partial genes and, therefore difficult to annotate. Another level of complexity results from gene insertion and/or deletion polymorphisms that are frequent in multigene families. As the genome assembly results from joined DNA fragments from different haplotypes, and from one or the other chromosome, it does not reflect a 'true' haplotype and a careful analysis is required for the gene and allele assignment. It should also be noted that some IG and TR genes, designated as orphons are localized outside the main loci [1,2]. Although these orphons are not functional, they have a high percentage of identity with genes of the major loci and represent another source of possible confusion and errors in gene and allele identification. At last, D and J genes have very small coding regions (8-37 base pairs (bp) and 37-69 bp, respectively) making their identification difficult.

Interestingly, and despite the difficulties mentioned above, functional IG and TR genes have characteristics that, if present, allow their unambiguous identification. Thus, for example, IG and TR V genes comprise two exons, a L-PART1 (L for leader) exon and a V-EXON with a splicing frame of type 1 (sf1) (IMGT Aide-mémoire, http://www.imgt.org/). The IG and TR V, D and J genes have recombination signals (RS) which allow them to rearrange at the DNA level in B cells (for the IG) and T cells (for the TR) [1,2] and that constitute one of the major differences with conventional genes. RS are localized in 3' of the V genes (V-RS), in 5' of the J genes (J-RS) and on both sides of the D genes (5'D-RS and 3'D-RS) [1,2]. They consist of conserved heptamers and nonamers separated by less conserved spacers of 12 ± 1 or 23 ± 1 bp which vary between loci and species (IMGT Repertoire, http://www.imgt.org/). An efficient recombination only occurs between a RS with a 12 bp spacer and a RS with a 23 bp spacer (12/23 rule) [29].

In IMGT^®^, the IG and TR gene characteristics used for gene identification are defined by concepts generated from the IMGT-ONTOLOGY 'IDENTIFICATION' axiom [9-11]. Three concept instances of the 'Molecule_EntityType' concept of identification are used in the IMGT/LIGMotif model: V-gene, D-gene and J-gene. These concept instances are defined by the gene type (variable (V), diversity (D), joining (J)), the molecule type (gDNA) and the configuration type (germline) [11].

### IG and TR gene characteristics for standardized gene description

#### Prototypes and labels

**Figure 1:**
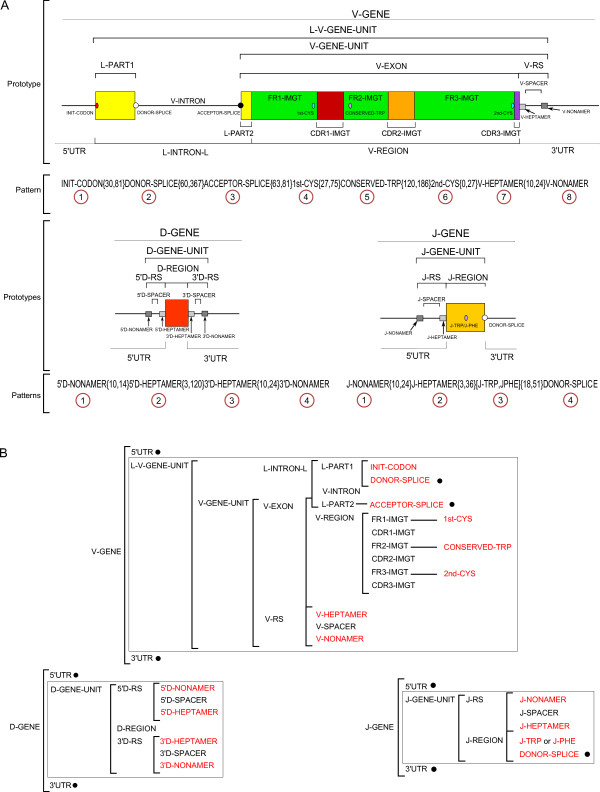
**Prototypes, labels and patterns**. A. V-GENE, D-GENE and J-GENE prototypes with labels and corresponding patterns. B. Labels for each prototype and gene unit. A L-V-GENE-UNIT is described with 24 labels (22 specific and 2 shared ones). A D-GENE-UNIT is described with 10 labels (all specific). A J-GENE-UNIT is described with 8 labels (7 specific labels, J-PHE and J-TRP being mutually exclusive and 1 shared one). Three additional labels, 1 specific and 2 common ones (5'UTR and 3'UTR), allow to describe the V-GENE (27 labels), D-GENE (13 labels) and J-GENE (11 labels). Shared and common labels are shown in black circle. Labels in red are conserved motifs.

The IG and TR gene features are described, in IMGT^®^, according to the standardized concepts of description generated from the IMGT-ONTOLOGY 'DESCRIPTION' axiom [9-11]. Thus, the V-gene, D-gene and J-gene are described by three concept instances of the 'Molecule_EntityPrototype' concept of description: V-GENE, D-GENE and J-GENE [11], respectively. Their graphical representation, or prototype, and the labels that describe them are shown in Figure 1 (A and B, respectively). Among the 242 IMGT^® ^labels defined for the nucleotide sequences, 47 are used in the IMGT/LIGMotif model (Figure 1B) of which 43 are specific of one prototype (23 for a V-GENE, 11 for a D-GENE and 9 for J-GENE). Two labels (5'UTR and 3'UTR) are common to all prototypes, whereas 2 labels (ACCEPTOR-SPLICE and DONOR-SPLICE) are shared by several IMGT-ONTOLOGY prototypes (shown with a black circle in Figure 1B).

The organization of a prototype is based on the relations that order two labels [11]. Interestingly, a set of twelve relations is necessary and sufficient to describe the relations between labels in a prototype (Table 1). Ten of these relations were defined previously [9-11]. Two reciprocal relations, 'is_in_5_prime_of' and 'is_in_3_prime_of' have been added in the IMGT/LIGMotif model to indicate the relative position of labels on a 5'-3' DNA strand when there is no intersection between labels (Table 1).

**Table 1:** IMGT-ONTOLOGY relations between labels.

Relation	Reciprocal relation
'adjacent_at_its_5_prime_to'	'adjacent_at_its_3_prime_to'
'included_with_same_5_prime_in'	'includes_with_same_5_prime'
'included_with_same_3_prime_in'	'includes_with_same_3_prime'
'overlaps_at_its_5_prime_with'	'overlaps_at_its_3_prime_with'
'included_in'	'includes'
'is_in_5_prime_of'	'is_in_3_prime_of'

Relations between labels are those used for the description of prototypes (graphical representation of instances of the 'Molecule_EntityPrototype' concept of description) in IMGT-ONTOLOGY [9-11].

#### Patterns

For the purpose of gene description, IMGT/LIGMotif uses the 'gene unit' labels L-V-GENE-UNIT, D-GENE-UNIT and J-GENE-UNIT (Figure 1A). Indeed, these labels, in contrast to the 'gene' labels (V-GENE, D-GENE and J-GENE) have the advantage to be precisely delimitated in 5' and 3', respectively, by the 5' end and 3' end of constitutive labels (L-PART1 and V-RS for V, 5'D-RS and 3'D-RS for D, and J-RS and J-REGION for J, respectively). Moreover, the part of the prototype they encompass can be defined by conserved motifs that constitute a pattern (Figure 1A). In a pattern, the conserved motifs are separated from each other by a distance in base pairs (bp) comprised between a minimal and a maximal length (between braces in Figure 1A). Motifs are ordered from 5' to 3' with a rank (in a circle in Figure 1A) that corresponds to their relative localization in the pattern, the motif the most in 3' having a rank that corresponds to the number of motifs in the pattern (that is 8 for V, 4 for D and 4 for J). In the J pattern, the motifs J-TRP and J-PHE are shown between brackets and separated with a coma to indicate that the two motifs are possible for the same rank.

These conserved amino acids J-TRP and J-PHE are part of a conserved motif '[W, F]-[G, A]-X-G' where W = tryptophan (J-TRP), F = phenylalanine (J-PHE), G = glycine, A = alanine and X = any amino acid except proline.

### IG and TR gene characteristics for functionality identification

The gene functionality identification can only be assigned to precisely described IG and TR genes. In IMGT-ONTOLOGY, an unrearranged genomic V, D or J gene can be functional (F), open reading frame (ORF) or pseudogene (P) [9]. A gene is qualified as 'functional' if the coding region has an open reading frame without stop codon, and if there is no described defect in the splicing sites, recombination signals and/or regulatory elements. A gene is qualified as 'ORF' if the coding region has an open reading frame, but alterations have been described in the splicing sites, recombination signals and/or regulatory elements and/or changes of conserved amino acids have been suggested by the authors to lead to uncorrect folding, and/or the entity is an orphon. A gene is qualified as 'pseudogene' if the coding region has stop codon(s) and/or frameshift mutation(s). In particular, a V-GENE (or V-GENE-UNIT) is considered as 'pseudogene' if these defects occur in the L-PART1 and/or V-EXON, or if there is a mutation in the L-PART1 INIT-CODON atg. A J-GENE (or J-GENE-UNIT) is considered as 'pseudogene' if it has been identified by the presence of a RS upstream of an open reading frame, but it has no donor splice site in 5' or the donor splice is not in the expected splicing frame sf1 or if it has no conserved '[W, F]-[G, A]-X-G' motif.

### Characteristics for gene delimitation and cluster assembly

The IMGT^® ^rule to delimit V-GENE, D-GENE and J-GENE instances is to equally distribute the distance between the two genes. The IMGT-ONTOLOGY 'GeneCluster' concept allows describing genomic sequences that contain several genes. The gene instances in a cluster can be of the same prototype (for example, a V-CLUSTER only contains V genes), or of different prototypes (for example, a V-D-J-CLUSTER contains at least one V gene, one D gene and one J gene). Seven instances of the 'GeneCluster' concept are used in the IMGT/LIGMotif model (Table 2). The IMGT-ONTOLOGY 'GeneCluster' instances are particularly useful for the annotation of the large scale genomic IG and TR loci and are also used by the Sequence Ontology (SO) [30] (Table 2).

**Table 2:** 'GeneCluster' concept instances used in IMGT/LIGMotif.

		'Molecule_EntityPrototype' concept instance	
		
IMGT-ONTOLOGY "GeneCluster" concept instance	Sequence Ontology	Minimal number of different instances	Name of the different instances
V-CLUSTER	SO:0000526	1	V-GENE
J-CLUSTER	SO:0000513	1	J-GENE
D-CLUSTER	SO:0000559	1	D-GENE
D-J-CLUSTER	SO:0000560	2	D-GENE
			J-GENE
V-D-CLUSTER		2	V-GENE
			D-GENE
V-J-CLUSTER	SO:0000534	2	V-GENE
			J-GENE
V-D-J-CLUSTER	SO:0000532	3	V-GENE
			D-GENE
			J-GENE

(1) Seven 'GeneCluster' concept instances of IMGT-ONTOLOGY are used in IMGT/LIGMotif. Six of them are also used by Sequence Ontology (SO) [30].

(2) Relations with the 'Molecule_EntityPrototype' concept instances comprise the minimal number of different instances for each 'GeneCluster' concept instance and the name of the different instances, as defined in the IMGT/LIGM-DB list of labels [31].

### IMGT/LIGMotif model

**Figure 2:**
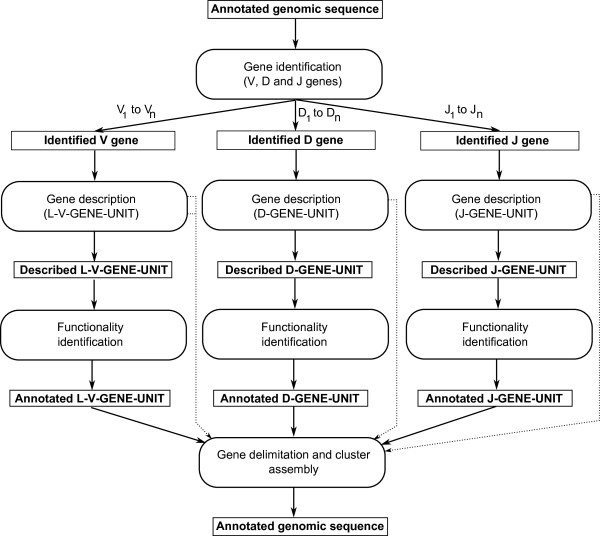
**IMGT/LIGMotif model overview**. The 4 modules IMGT/LIGMotif comprise 'Gene identification', 'Gene description', 'Functionality identification' and 'Gene delimitation and cluster assembly'. Dots indicate partially described and undescribed outputs (for V, D and J) of the 'gene description' module that are entered, for complete analysis of the sequence to analyse, in the last module.

The IMGT/LIGMotif model comprises 4 modules (Figure 2), 3 of them ('Gene identification', 'Gene description' and 'Functionality identification') take into account the IG and TR gene characteristics as defined above and deal with individual gene units, whereas the fourth module ('Gene delimitation and cluster assembly') deals with gene delimitation and assembly of genes in a cluster, and provides an annotated genomic sequence.

#### Gene identification

The 'Gene identification' module identifies potential V, D and J genes along the genomic sequence to analyse. First, a heuristic search for local alignments is performed against IMGT/LIGMotif reference motif databases (Table 3). These databases comprise nucleotide sequences that correspond to IG and TR gene unit labels (L-V-GENE-UNIT, D-GENE-UNIT, J-GENE-UNIT) and to motifs that compose them. These databases are created dynamically from IMGT/LIGM-DB [31] sequences, using the IMGT/GENE-DB [16] interface that allows queries on labels. Thirty-four labels were queried corresponding to IG and TR sequences from human (*Homo sapiens*) and mouse (*Mus musculus*; few sequences were also included from *Mus pahari*, *Mus saxicola*, *Mus spretus*). Pseudogene genes too poorly conserved to be assigned to subgroups were excluded. The alignments obtained in this first step provide labelled high-scoring segment pairs (or HSPs) on both DNA strands of the sequence to analyse. Then, there is a selection of the labelled HSPs and a grouping of these selected HSPs given their topology and their gene type (V, D or J). Thus, the 'Gene identification' module provides the potential V genes, D genes and J genes identified as grouped and labelled HSPs along the sequence to analyse.

**Table 3:** IMGT/LIGMotif reference motif databases.

			Number of sequences			
					
Prototype	Number of databases	Reference motif databases	Human	Mouse	Gene identification (Blast HSPs)	Gene description and functionality identification
V-GENE	16	L-V-GENE-UNIT	368	204	+	
		V-GENE-UNIT	385	221	+	
		L-PART1	534	550	+	
		V-INTRON	575	518	+	
		L-INTRON-L	470	288	+	
		V-EXON	660	550	+	+
		L-PART2	591	580	+	
		V-REGION	887	1036	+	
		FR1-IMGT	785	730	+	
		CDR1-IMGT	790	731	+	
		FR2-IMGT	790	739	+	
		CDR2-IMGT	788	737	+	
		FR3-IMGT	788	737	+	
		CDR3-IMGT	674	607	+	
		V-RS	378	222	+	
		V-SPACER	485	449	+	
	
	2	V-HEPTAMER	326	317		+
		V-NONAMER	262	298		+

D-GENE	6	D-GENE-UNIT	36	28	+	
		5'D-RS	40	29	+	
		5'D-SPACER	50	29	+	
		D-REGION	50	38	+	
		3'D-RS	36	32	+	
		3'D-SPACER	47	32	+	
	
	4	5'D-NONAMER	36	22		+
		5'D-HEPTAMER	36	22		+
		3'D-HEPTAMER	36	21		+
		3'D-NONAMER	33	21		+

J-GENE	4	J-GENE-UNIT	120	107	+	
		J-RS	120	107	+	
		J-SPACER	120	108	+	
		J-REGION	130	121	+	
	
	2	J-NONAMER	101	76		+
		J-HEPTAMER	101	77		+

#### Gene description

The 'Gene description' module provides the description of each potential gene identified in the first module. It comprises a search of conserved motifs based on prototypes and patterns. Codons of conserved amino acids in the patterns ('tgg' for CONSERVED-TRP and J-TRP, 'tgc' and 'tgt' for 1st-CYS and 2nd-CYS, and 'ttt' and 'ttc' for J-PHE) are difficult to identify by conventional algorithms as the motifs (triplets 'tgg', 'tgc', 'tgt', 'ttt' and 'ttc') are very frequent in sequences. For that reason, the codons of conserved amino acids of the V-REGION and V-EXON (that comprise 1st-CYS, CONSERVED-TRP and 2nd-CYS) are identified by the software IMGT/V-QUEST [32]. The expected outputs of the 'Gene description' module are described as gene units (GENE-UNIT), although, as discussed in the algorithm section, partially described and undescribed outputs can also be obtained.

#### Functionality identification

The 'Functionality identification' module includes the control of features needed for the functionality assignment and allows to obtain annotated gene units.

#### Gene delimitation and cluster assembly

In this final module, genes (V-GENE, D-GENE and J-GENE) are delimited and assembled in a cluster if the analysed genomic sequence contains several genes. The final outcome of IMGT/LIGMotif is the annotated genomic sequence.

## Algorithm

### Gene identification of V, D and J genes

#### Search of labelled alignments

**Figure 3:**
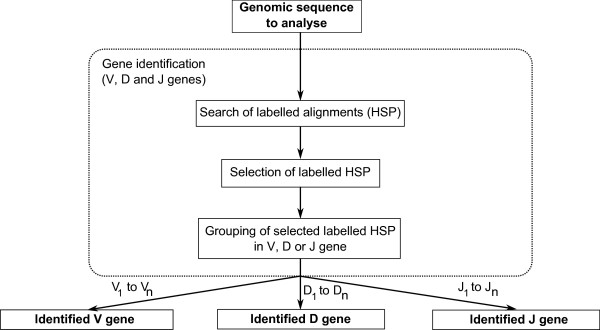
**IMGT/LIGMotif 'Gene identification' module**. The output of the 'Gene identification' module is an 'Identified V gene', 'Identified D gene' and 'Identified J gene'. Each gene is identified by group of labelled HSPs selected on their BLAST parameters.

The algorithm starts by aligning the genomic sequence using BLASTN [33] against IMGT/LIGMotif reference motif databases (Table 3, Figure 3). The possibility is given to the biocurator to select databases on species (human and/or mouse), locus (IGH, IGK, IGL, TRA, TRB, TRG and/or TRD), gene type (V, D and/or J), functionality (F, ORF and/or P), and to choose any combination in this selection. IMGT/BLAST provides HSPs that inform on the similarity of the analysed sequence (query) with labelled motifs from the reference database (subject). These labelled HSPs are obtained on both DNA strands of the sequence to analyse. Practically, NCBI-BLASTN (version 2.2.18) is used with the minimum hit word size possible (i.e. 4), an E-value threshold of 0.01 (except for D-GENE-UNIT and its motifs where an E-value threshold of 5 is selected owing to their very short length). The BLAST was preferred to Hidden Markov Model (HMM) methods and software such as HMMER [34,35] for practical uses.

#### Selection of labelled HSPs

IMGT/BLAST produces a huge quantity of HSPs but these HSPs do not have the same importance. HSPs obtained with different reference motif databases may overlap at a same location as they are expected components of a same prototype (e.g. V-EXON overlaps V-REGION). These overlapping HSPs do not need to be filtered as they delimit different and expected labels. In contrast, and owing to gene duplications in IG and TR loci, different HSPs obtained with a same reference motif database may overlap at a same location although they might belong to different genes. In consequence, the best HSPs are selected at a given location (Figure 3) on score, E-value, length and identity BLAST parameters. The method to filter overlapping HSPs obtained with the same motif database is described as follows: score(), length(), identity(), evalue() are the functions that return 1 if a given HSP (hsp1) performs better than the other (hsp2) for the tested parameter (higher score, length, identity, and lower E-value), 0 if the two HSPs are equal and -1 if hsp1 performs less well than hsp2. Parameter g1 (sum of score() and evalue(), with -2 ≤ g1 ≤ 2) and parameter g2 (sum of length() and identity(), with -2 ≤ g2 ≤ 2) are calculated between each two overlapping HSPs obtained from the same motif database. If g1 > 0, or if g1 = 0 and g2 > 0, hsp2 is removed. In other cases (g1 < 0, or g1 = 0 and g2 < 0), hsp1 is removed.

#### Grouping of selected HSPs in V, D or J gene

**Figure 4:**
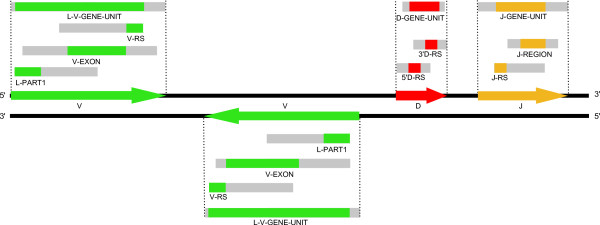
**Grouping of selected labelled HSPs into V, D and J genes**. Selected labelled HSPs are grouped into V, D and J genes, respectively. The rectangles in grey represent the length added in 5' and or 3' of the selected HSPs. A search of position overlaps with the other selected and extended HSPs allowed the grouping. Arrows indicate the gene orientation relative to the DNA strand, as deduced by the algorithm by the respective positions of the labelled HSPs. Note that the labels at this stage refer to labelled HSPs and gene identification, and not to a detailed description of the genes.

The objective of this step is to group selected HSPs that may belong to a same gene (Figure 3). For that purpose, the positions of selected HSPs from the same DNA strand and with labels of the same gene type (V, D or J) are, in this step, compared to each other (Figure 4). If the topological relation of two compared HSPs is not coherent they are considered as belonging to distinct genes. If the topological relation is coherent, a length specific for each label (Table 4), is added to 5' and/or 3' extremities of each compared HSPs (Figure 4) and these new regions are looked for a potential position overlap. If a position overlap is found, the two HSPs are considered as belonging to the same gene. If not, the two HSPs are considered as belonging to different genes. For a given HSP group, the position most in 5' and the position most in 3' define the area that contains a potential gene (shown as arrows in Figure 4). The labelled HSPs that constitute that group provide the gene type (V, D or J) and, using their respective localization on the DNA strands, the gene orientation. Each group of HSPs corresponds to a potential gene identified along the sequence (indicated with V_1 _to V_n_, D_1 _to D_n _and J_1 _to J_n _in Figure 3).

**Table 4:** Lengths (in nucleotides) added to HSP extremities.

Gene type	V				D	J
	**L-PART1**	**V-REGION**	**FR1-IMGT**	**V-SPACER**	**D-GENE-UNIT**	**J-GENE-UNIT**
		**V-EXON**	**FR2-IMGT**	**V-RS**	**5'D-RS**	**J-RS**
**Label**			**FR3-IMGT**		**5'D-SPACER**	**J-SPACER**
					**D-REGION**	**J-REGION**
					**3'D-RS**	
					**3'D-SPACER**	

3' end	0	500	300	0	58	30

5' end	500	500	300	500	58	30

### Gene description of L-V-GENE-UNIT, D-GENE-UNIT and J-GENE-UNIT

**Figure 5:**
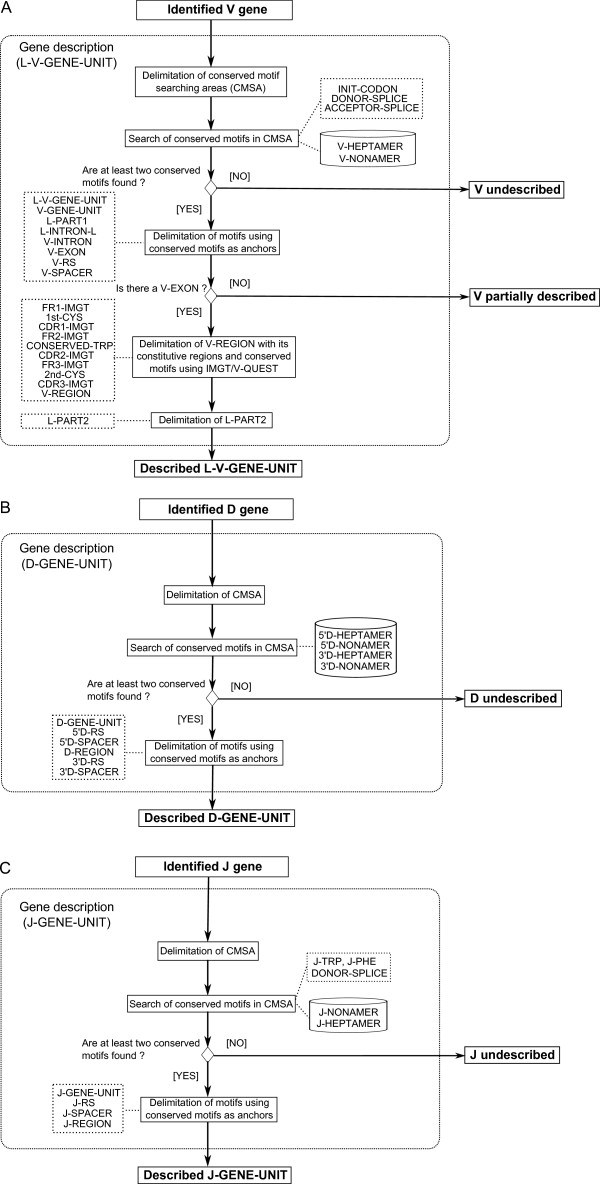
**IMGT/LIGMotif 'Gene description' module**. The output of the 'Gene description' module is a 'Described V-GENE-UNIT' (A), 'Described D-GENE-UNIT' (B) or 'Described J-GENE-UNIT' (C). The delimitation of conserved motif searching areas (CMSA) is described in the text. At least two conserved motifs need to be found. If not, the output is 'V undescribed', 'D undescribed' and 'J undescribed'. The absence of V-EXON for a V gene leads to a 'V partially described'. The delimitation of motifs using conserved motifs as anchors is shown in Figure 6.

The second module of IMGT/LIGMotif, 'Gene description', describes in detail each identified gene, individually (Figure 5). This analysis is performed from 5' to 3', exploring both strands. IMGT/LIGMotif starts by searching conserved motifs of the patterns described in Figure 1A. The gene description is performed by searching and delimiting conserved motifs that are characteristic of each gene type (V, D and J).

#### Delimitation of conserved motif searching areas (CMSA)

In order to reduce the algorithm execution time the search of conserved motifs is limited to conserved motif searching areas (or CMSA) which are delimited by the positions of the most informative combination in each grouped HSP. The best combination of HSPs depends on the gene type (Table 5): for instance, for a V gene the best combination is L-PART1+V-EXON+V-RS, for a D gene, it is 5'D-RS + 3'D-RS, whereas for a J gene, it is J-RS + J-REGION. If the best combination is not present, other combinations are explored in the order shown in Table 5. A length is added to extremities (5' and/or 3') of the labels belonging to an HSP combination to delimit the CMSA (Table 6). For example, a length of 40 nt (the maximum length of a RS) is added to the 5' and 3' position ends of a RS HSP.

**Table 5:** Combinations of labelled HSPs used for the delimitation of conserved motif searching areas (CMSA).

Prototype	L-V-GENE-UNIT	D-GENE-UNIT	J-GENE-UNIT
Combination of labelled HSPs	L-PART1 + V-EXON + V-RS L-PART1 + V-EXON V-EXON^(2) ^+ V-RS V-EXON^(2)^ L-V-GENE-UNIT	5'D-RS + 3'D-RS^(1)^ 5'D-RS + D-REGION D-REGION + 3'D-RS 5'D-RS 3'D-RS D-REGION D-GENE-UNIT	J-RS + J-REGION J-REGION J-RS J-GENE-UNIT

The underlined combinations are those used in priority. If not present, the combinations below in the columns are chosen in the order from top to bottom.

(1) D-REGION is not used even if it is identified because 5'D-RS and 3'D-RS are sufficient for its precise delimitation, as well as those of the heptamers and nonamers.

(2) As L-PART1 is missing, its motifs (INIT-CODON and DONOR-SPLICE) are not delimited.

**Table 6:** Lengths (in nucleotides) used for the delimitation of conserved motif searching areas (CMSA).

Gene type	Labels	Length
V	L-PART1	90
	V-EXON	375
	V-RS	40

D	D-GENE-UNIT	200
	D-REGION	120
	5'D-RS	40
	3'D-RS	40

J	J-GENE-UNIT	160
	J-REGION	120
	J-RS	40

#### Search of conserved motifs in CMSA

Conserved motifs that include conserved amino acids (INIT-CODON for V, J-TRP and J-PHE for J), splicing sites (DONOR-SPLICE, ACCEPTOR-SPLICE), heptamers and nonamers are searched in the CMSA which are known to include them. Heptamers and nonamers are searched by alignment with the reference motif databases. If no exact match is found, an approximate form of the motifs is searched using non gapped position-specific scoring matrices (PSSM) [36]. Following the search of conserved motifs in CMSA, matches are grouped and retained as a set named 'solution' if the distances between the motifs are in the intervals defined for the pattern. A minimum of two conserved motifs is required to retain the solution. If this condition is not fulfilled, genes cannot be described using IMGT/LIGMotif and, thus, are defined as 'V undescribed', 'D undescribed' and 'J undescribed' (Figure 5).

#### Delimitation of motifs using conserved motifs as anchors

**Figure 6:**
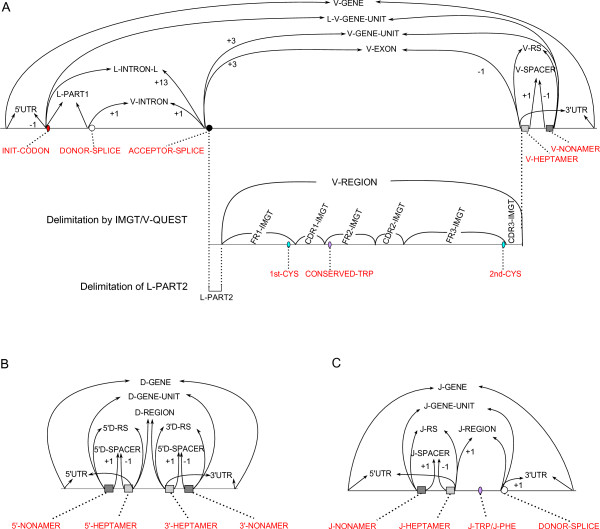
**Delimitation of motifs using conserved motifs as anchors**. The delimitation of motifs using conserved motifs as anchors is used for the description of a V-GENE-UNIT (A), D-GENE-UNIT (B) and J-GENE-UNIT (C). This approach is necessary and sufficient for the description of a D-GENE-UNIT or that of a J-GENE-UNIT. The description of a V-GENE-UNIT requires two additional steps: the delimitation of the V-REGION and of its constitutive regions and conserved motifs by IMGT/V-QUEST [32], and the delimitation of L-PART2.

In the next step, other motifs of the patterns are then delimited precisely using conserved motifs as anchors (or seeds) (Figure 6). Arrows taking root from anchors delimit precisely new labelled motifs. For instance, ACCEPTOR-SPLICE and V-HEPTAMER (conserved motifs) allow delimiting V-EXON. An arrow arriving from the left delimits the 5' end of a motif whereas an arrow arriving from the right delimits the 3' end. The number associated to an arrow indicates the number of nucleotides which must be added (+) or subtracted (-) to a conserved motif position to delimit precisely the new labelled motif. The J-PHE and J-TRP are the only conserved motifs that do not delimit labelled motifs, and consequently, no arrow takes root from them.

#### Additional steps for the description of a L-V-GENE-UNIT

The description of a L-V-GENE-UNIT requires two additional steps. The first one is the delimitation of the V-REGION with its constitutive regions (FR-IMGT and CDR-IMGT) and conserved motifs (1st-CYS, CONSERVED-TRP and 2nd-CYS) using IMGT/V-QUEST [32]. This step is only be performed if a V-EXON has been identified. If V-EXON is missing the gene is defined as 'V partially described'. The final step for the description of a L-V-GENE-UNIT is the delimitation of L-PART2, a region delimited by the V-EXON acceptor splice and the 5' end of the V-REGION determined by IMGT/V-QUEST.

### Functionality identification

**Figure 7:**
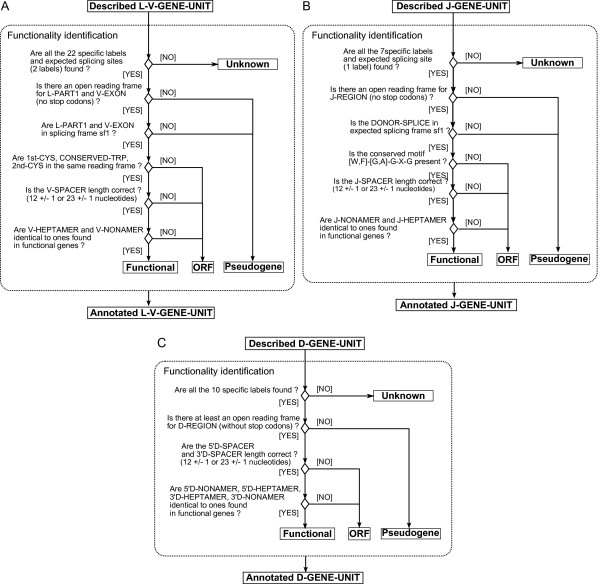
**Functionality identification**. The output of the 'Functionality identification' module is 'Annotated L-V-GENE-UNIT' (A), 'Annotated J-GENE-UNIT' (B) and 'Annotated D-GENE-UNIT' (C). The identification of the functionality is 'Functional', 'ORF' and 'Pseudogene'. If the first criterion is not fulfilled, the functionality is 'Unknown' as it cannot be identified automatically. In the figure, heptamers and nonamers are quoted from 5' to 3' relative to gene units.

The third module of IMGT/LIGMotif 'Functionality identification' identifies the functionality of each described gene unit (Figure 7). A L-V-GENE-UNIT is identified as functional if it has all the 22 specific labels and expected splicing sites (2 labels), no stop codons in L-PART1 and V-EXON, a splicing frame sf1 between L-PART1 and V-EXON, no frameshift (same reading frame for 1st-CYS, CONSERVED-TRP and 2nd-CYS), an expected V-SPACER length, and V-HEPTAMER and V-NONAMER identical to ones found in functional genes (Figure 7A). A J-GENE-UNIT is identified as functional if it has all the 7 specific labels and expected splicing site (1 label), no stop codons in J-REGION, a sf1 donor splice, the conserved '[W, F]-[G, A]-X-G' motif as one indicator of the absence of frameshift, an expected J-SPACER length, and J-NONAMER and J-HEPTAMER identical to ones found in functional genes (Figure 7B). A D-GENE-UNIT is identified as functional if it has all the 10 specific labels, at least one open reading frame without stop codon(s), expected 5'D-SPACER and 3'D-SPACER lengths, and the heptamers and nonamers are identical to ones found in functional genes (Figure 7C). A gene unit is identified as an open reading frame (ORF) if the last 3 criteria for V and J and the last 2 for D are not fulfilled. In other cases the gene unit is identified as a pseudogene (P). Note that if the first criterion (number of specific labels and splicing sites) is not fulfilled, the functionality is 'Unknown' as it cannot be determined automatically and its identification therefore requires a manual expertise.

### Gene delimitation and cluster assembly

For each gene unit, and whatever its description status (that is either annotated (L-V-GENE-UNIT, D-GENE-UNIT, J-GENE-UNIT), partially described ('V partially described') or undescribed ('V undescribed', 'D undescribed', 'J undescribed')), the 5'UTR and 3'UTR delimitations of the corresponding V-GENE, D-GENE or J-GENE are determined by equally distributing the distance between two neighbouring gene units. Thus, the sequence can be considered as a succession of genes that constitute a cluster. The cluster is defined based on the 'Molecule_EntityPrototype' instances found in the sequence and an IMGT^® ^cluster label is assigned (Table 2), for instance V-D-J-CLUSTER, if the sequence contains at least one V-GENE, one D-GENE and one J-GENE. Finally, the number of genes in the analysed sequence is computed per DNA strand, per gene type and per functionality.

## Results and Discussion

**Figure 8:**
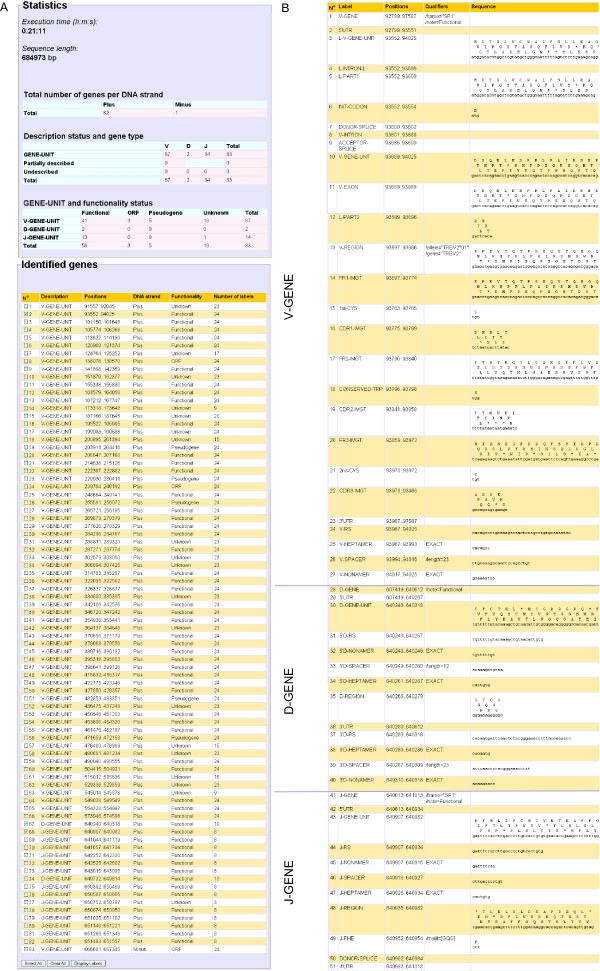
**IMGT/LIGMotif result page**. A. Statistics and Identified genes. The main table provides the identified genes from the 5' end to the 3' end of the DNA sequence (from top to bottom of the table). Three genes were selected to display their labels: a V-GENE-UNIT (N° 2, a D-GENE-UNIT (N° 58) and a J-GENE-UNIT (N° 59). B. Display of labels for the genes selected in the main table.

IMGT/LIGMotif algorithm is implemented in JAVA http://www.java.com/fr/. A web application of IMGT/LIGMotif is running on a Tomcat server http://tomcat.apache.org/ and is available at http://www.imgt.org/ligmotif/. The genomic sequence to analyse can be copied/pasted by the biocurators, or uploaded in Fasta or EMBL format. Query parameters can be modified to optimise the analysis efficiency. For instance, reference motif databases can be selected on their gene type, locus, functionality and organism. The execution time depends on the gene types and the number of genes existing in the sequence. The analysis of a single gene takes a few seconds whereas a complete locus containing more than 100 genes takes between 30 minutes to 1 hour, using standard parameters. The IMGT/LIGMotif results page (Figure 8) displays, at the top of the page, statistics that include the execution time, the length of the analysed sequence, the total number of genes per DNA strand (plus or minus), and two tables: the first one indicates the number of genes per description status (GENE-UNIT, Partially described, Undescribed) and per gene type, the second table indicates the number of annotated GENE UNIT per functionality status (Functional, ORF, Pseudogene, Unknown). Below the statistics, the main table displays the content of the analysed sequence, starting from N° 1 (in column 1) for the gene the most in 5'. This table provides, for each identified gene: Description (GENE-UNIT label or description status), Positions (in the analysed sequence), DNA strand, Functionality and Number of labels. Selecting gene units in the main table (Figure 8A) allows displaying their labels with positions, nucleotide sequences and, for coding regions, amino acid sequences (Figure 8B). In the detailed display, labels of each gene unit are ordered from 5' to 3'. If two labels start at the same 5' position, the label with the longest nucleotide length is displayed first. The IMGT/LIGMotif results can be exported to a spreadsheet file that can be modified at will. IMGT/LIGMotif results for the 684,973 bp sequence of the human TRB locus (L36092 of IMGT/LIGM-DB [31]) identified 83 gene units (67 V, 2 D and 14 J) (Figure 8). Compared with manual expert annotation, these results show that all gene units were correctly identified. No gene was missing and there was no false positive. The functionality is more difficult to assign, which is reflected in the IMGT/LIGMotif results. Functionality could be assigned to 64 gene units: 56 genes (41 V, 2D and 13 J) were determined as functional, 3 as ORF and 5 as pseudogenes. Indeed, the functionality identification by IMGT/LIGMotif requires that a gene unit be fully described with all its labels. Most mutated pseudogenes have missing labels therefore preventing the functionality assignment. Another limitation is the lack of recognition of unconventional splicing sites found in ORF (for example a donor splice with the sequence 'nag' instead of 'ngt'). Although manual expertise is still required for a definitive functionality assignment, IMGT/LIGMotif represents a major step forward in the automatic assignment of the functionality. In its present status, IMGT/LIGMotif considerably accelerates the process of identification and description of antigen receptor genes in large genomic sequences.

## Conclusions

IMGT/LIGMotif http://www.imgt.org/ligmotif/ is a user friendly tool that provides the annotation of large genomic sequences containing IG and TR genes. The web user interface provides a simple way to query, visualize and download results. The execution time is suitable for the analysis of an entire locus. The annotation includes the gene identification and orientation in the sequence, the gene delimitation (V-GENE, D-GENE and J-GENE), the detailed description of gene units (L-V-GENE-UNIT, D-GENE-UNIT and J-GENE-UNIT) with the IMGT^® ^labels and functionality identification, and finally the cluster assembly. Annotation of C-GENE has not been included in this first version of IMGT/LIGMotif as the gene identification and annotation is performed easily with conventional tools by the biocurators. IMGT/LIGMotif is particularly useful for the annotation of IG and TR loci from species that are phylogenetically close to human and mouse such as nonhuman primate species, chimpanzee and rat, respectively. More distant species will still require manual expertise in the control of the annotations. However, it is expected that the progressive enrichment of the IMGT/LIGMotif reference motif databases with data IG and TR annotated by IMGT^® ^will save a considerable amount of time in the process of the genomic annotation of vertebrate antigen receptor loci.

## Competing interests

The authors declare that they have no competing interests.

## Authors' contributions

JL conceived the algorithm and its implementation. PD and MPL coordinated the project. All authors read and agree to publish the manuscript.
